# Rosette margination in blood flow during malaria pathogenesis

**DOI:** 10.1016/j.bpj.2025.12.023

**Published:** 2025-12-22

**Authors:** Anil Kumar Dasanna, Marianne Papagrigorakes, Michael Lanzer, Ulrich S. Schwarz, Gerhard Gompper, Dmitry A. Fedosov

**Affiliations:** 1 Theoretical Physics of Living Matter, Institute for Advanced Simulation, Forschungszentrum Jülich, 52425 Jülich, Germany; 2 Department of Physical Sciences, Indian Institute of Science Education and Research (IISER) Mohali, Sector 81, Knowledge City, Mohali 140306, India; 3 Department of Infectious Diseases, Heidelberg University, Heidelberg, Germany; 4 BioQuant-Center for Quantitative Biology and Institute of Theoretical Physics, Heidelberg University, Heidelberg, Germany

## Abstract

Margination is a physical phenomenon that describes the migration of cells and particles toward vessel walls in blood flow, and thus, it serves as a necessary precondition for the adhesion of particles suspended in blood plasma to the endothelium. In the context of malaria, adhesion of infected red blood cells (iRBCs) to the endothelium is essential for the disease progression, as iRBCs have to evade the removal from the blood circulation in the spleen. Some malaria strains lead to the formation of rosettes, multicellular structures composed of one iRBC surrounded by several adhered healthy RBCs (hRBCs). We employ mesoscopic hydrodynamics simulations and microfluidic experiments to investigate the margination of rosettes in blood flow at various flow rates, volume fractions of hRBCs, and binding strengths between iRBCs and hRBCs. Surprisingly, rosette margination is significantly weaker than that of single iRBCs, suggesting their limited adhesion potential in blood flow. The main reason for poor margination of rosettes is the deformability and dynamics of rosette clusters formed by several hRBCs around one iRBC. Simulation predictions are confirmed by microfluidic experiments. Our results suggest that the main function of rosette formation is not to enhance cytoadhesion to the endothelium, but to keep the iRBCs in the blood flow, at least along straight vessel segments.

## Significance

An important element of the blood stage of a malaria infection is the adhesion of parasite-infected RBCs (iRBCs) to the vascular endothelium. The cytoadhesion of iRBCs increases residency time in the vasculature and avoids removal during passage through the spleen. In addition to adhesion to the endothelium, some strains of malaria parasites cause iRBCs to bind to healthy RBCs, forming multicellular clusters called rosettes. Like cytoadhesion, the formation of rosettes is positively correlated with the severity of disease, as it also increases microvascular obstruction. Its main function is believed to be protection of the iRBCs from the immune system. We employ a combination of hydrodynamic simulations and microfluidic experiments to study the localization of rosettes in microvascular flow. Surprisingly, we find that rosette formation does not enhance cytoadhesion to the endothelium, as rosettes primarily remain near the center of blood vessels. Thus, our results suggest a reduced cytoadhesion for malaria strains with rosette formation.

## Introduction

Malaria is caused by *Plasmodium* parasites, whose development, multiplication, and transmission form a complex life cycle, involving both mosquito and human hosts (1,2). The medically most interesting stage of the life cycle is the blood stage of infection in a human host, because it is a symptomatic stage. The blood stage starts with merozoites released into the blood stream after the rupture of infected hepatocytes. In the blood circulation, merozoites invade healthy red blood cells (hRBCs) and develop and multiply inside the host cells. Parasite development within infected RBCs (iRBCs) proceeds in three stages, namely ring, trophozoite, and schizont stages, during which progressive changes in morphology and adhesion properties of iRBCs occur. At the end of the ring stage, iRBCs develop adhesion patches (or knobs) at the membrane surface with a typical size of 100 nm (3), which has been visualized by both scanning electron microscopy (4) and atomic force microscopy (5). As a result, iRBCs in the trophozoite and schizont stages start to adhere to vascular endothelium (1,6). Other important host-cell modifications by the parasite are changes in the morphology and stiffness of iRBCs. The cell shape changes from discocyte at the ring stage, to oval at the trophozoite stage, and to near spherical at the schizont stage (7,8). At the same time, the stiffness of iRBCs increases by up to 10-fold (9). Finally, after about 48 h of the intraerythrocytic parasite development, iRBCs rupture, releasing about 20 new merozoites into the blood stream to continue the infection process (10).

**Figure 1:**
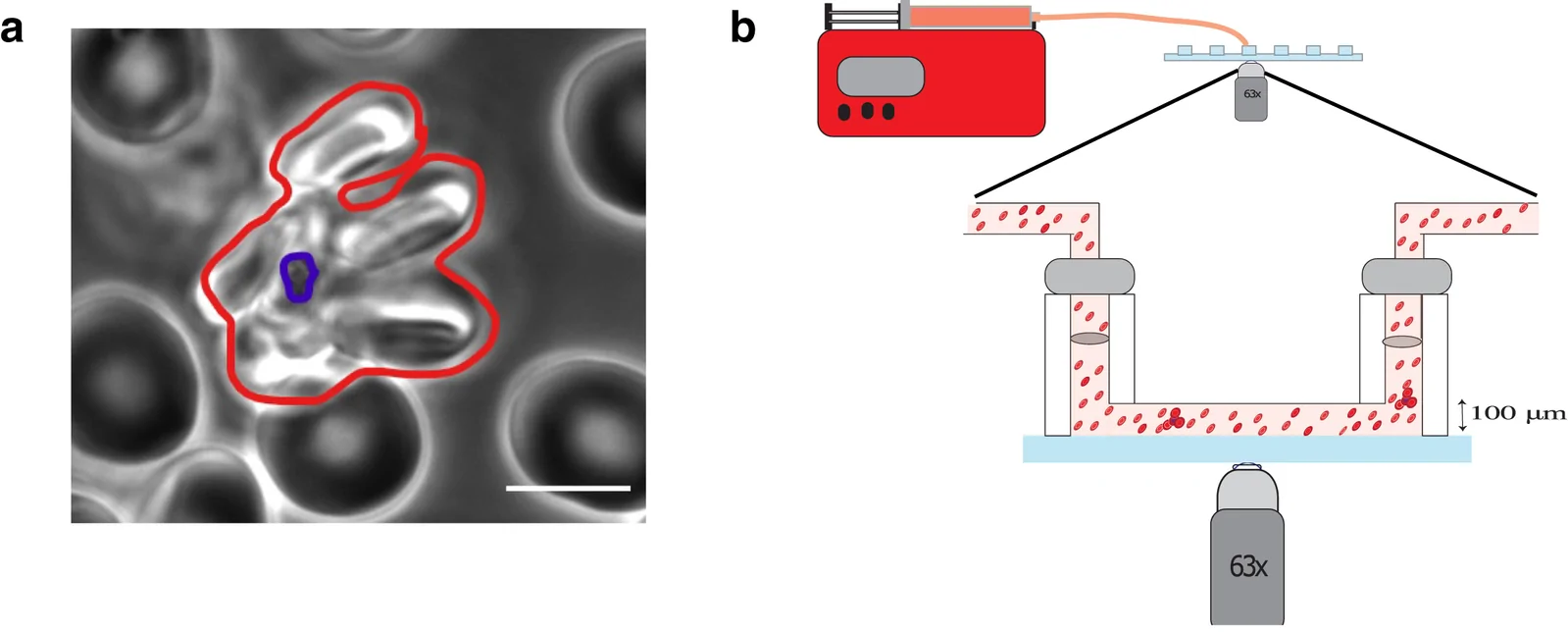
Rosette image and microfluidic setup for margination measurements. (*a*) A bright-field image of a rosette formed by one iRBC and several adhered hRBCs. The rosette is highlighted with red color, whereas the parasite nucleus is blue. See also Video S1. (*b*) Sketch of a microfluidic setup that has been used to measure rosette margination in experiments. The channel dimensions are as follows: length is 17 mm, width is 1 mm, and height (*W*) is 0.1 mm.

For some parasite strains, iRBCs can also adhere to surrounding hRBCs, resulting in multicellular clusters called rosettes (1). A bright-field image of a rosette is shown in Fig. 1 *a*. The clusters of one iRBC covered by several hRBCs are hypothesized to aid parasites in the evasion of the immune system and to facilitate rapid entry of released merozoites into the new host cells. However, there is no conclusive evidence to directly support either of these hypotheses. Several studies (11,12,13) link the formation of rosettes to the cases of severe malaria, in particular to those associated with cerebral malaria. Formation of rosettes has also been shown to increase microvascular obstruction, which significantly contributes to the severity and complications of malaria (14). *Plasmodium falciparum* isolates from patients with severe malaria have been shown to have a stronger tendency to rosetting, in comparison to isolates from patients with moderate illness (11,12,13). A few studies indicate a correlation between the formation of rosettes and the blood group. The blood group ABO has been demonstrated to favor the formation of rosettes when compared with the blood group O (15,16). For the O blood group, adhesive interactions within rosettes are mediated by *P. falciparum* erythrocyte membrane protein 1 (PfEMP-1) at the adhesive knobs, whereas for the ABO group, the formation of rosettes is facilitated by repetitive interspersed families of polypeptides (RIFINs) (17,18).

Video S1. Experimental observation of rosettes at low flow rates, where an aggregate of several RBCs is clearly visible

The adhesion of iRBCs to vascular endothelium is essential for the disease progression, because it prevents the passage of iRBCs through the spleen, where they have to squeeze through interendothelial slits and are removed by the immune system upon changes in their mechanical properties. Thus, iRBC adhesion at some microvascular location allows the parasites to fully develop and release new parasites after membrane rupture. iRBC adhesion to the endothelium is mediated by PfEMP-1 proteins located at the knobs, which have multiple binding partners (e.g., ICAM-1, CSA, CD-36) at the endothelial cells (19). Adhesion dynamics of iRBCs in flow strongly depends on the primary type of receptors. For instance, bindings between PfEMP-1 and CD-36 behave like slip bonds (i.e., bond dissociation probability increases with increasing applied stress), whereas bindings between PfEMP-1 and ICAM-1 or CSA behave like catch bonds (i.e., bond dissociation probability decreases with increasing applied stress) (20,21). Numerous computational investigations have quantified the adhesion dynamics of iRBCs on a substrate (22,23,24,25,26). Despite the fact that similar proteins mediate rosette formation, the importance of adhesion in this case is much less clear. Furthermore, most investigations of cytoadhesion under flow in malaria do not address the fact that iRBCs have to first reach vascular walls before any adhesion can take place.

The migration of certain cell types (e.g., white blood cells (WBCs)) (27,28,29) or drug delivery particles (30,31,32) toward vessel walls in blood flow is referred to as margination. Margination results from the interplay between hydrodynamic forces and deformability of the different components of blood, in particular, the three blood cell types (RBCs, WBCs, and platelets) (33,34). Deformable hRBCs are generally driven by lift forces to the vessel center, resulting in the localization of other components (mainly platelets) near vessel walls. Margination of single iRBCs has been investigated in several studies (35,36,37) and is attributed to the changes toward a spherical shape and an increased stiffness of iRBCs. In fact, iRBC margination compares well with the margination of WBCs in blood flow (38,39,40), though WBCs are generally larger in size than iRBCs. An interesting question in the context of rosette behavior in blood flow is how well rosette clusters marginate, since margination is a necessary precondition for their adhesion to the endothelium.

In this work, we study the margination propensity of rosettes in blood flow, using two-dimensional mesoscopic computer simulations and microfluidic experiments. Several important parameters for rosette margination are considered, including the rate of blood flow, hematocrit (volume fraction of hRBCs), and the strength of adhesive interactions between iRBCs and hRBCs. Strong adhesive interactions lead to stable rosettes (i.e., with a fixed composition of hRBCs adhered to a single iRBC) in flow, whereas weak adhesion results in dynamic rosettes (i.e., dynamic exchange of hRBC within the rosette cluster) or even single iRBCs, when adhesive interactions are small in comparison to stresses exerted by the flow. Surprisingly, our results show that stable rosettes have poor margination and generally flow near the channel center. With a decrease in the adhesive interactions between iRBCs and hRBCs, the margination propensity increases, yielding the best margination properties for single iRBCs. As a result, the poor margination of rosette clusters is expected to significantly reduce their adhesion to vascular endothelium.

## Materials and methods

### Flow chamber experiments

#### Rosetting culture

A *P. falciparum* strain that forms clusters with hRBCs (rosettes) has been provided by Alexandra Rowe from Edinburgh University. It was cultured in HbA erythrocytes, where the cultures were maintained at 3.5% hematocrit with no higher than 5% parasitemia. RPMI-1640 (Gibco) culture medium was supplemented with 2 mM L-glutamine, 20 mM glucose, 25 mM HEPES (Gibco), 20 *μ*g/mL gentamycin, 100 *μ*M hypoxanthine, 5% v/v A+ human serum, and 0.25% v/v Albumax (Gibco). Cultures were grown at 37°C under controlled atmospheric conditions (3% CO_2_, 5% O_2_, 92% *N*_2_, 96% humidity). Parasite maturity was assessed by microscope, with fixed thin blood smears stained with 10% Giemsa staining. Early-stage parasites were selected using the sorbitol lysis protocol, allowing for a close synchronization of cultures. Gelatin sedimentation protocol was performed to separate knobby nonrosetting parasites (supernatant) from rosetting parasites and hRBCs (pellet). This protocol was performed regularly to ensure a high rosetting probability.

#### Rosetting probability

A rosette is defined as at least one iRBC that adheres firmly to two or more hRBCs. Rosetting probability was measured using a wet slide observed through a light microscope at 50× magnification by counting at least 500 iRBCs. The percentage of those iRBCs forming a rosette was calculated, leading to the rosetting probability.

#### Flow assay

The experimental setup is illustrated in Fig. 1 *b*. Flow assay measurements were performed in a TCP parallel flow chamber (*μ*-slide VI 0.1, Ibidi) glued to a high-resolution glass slide (#1.5, ROTH) using freshly prepared and de-gassed PDMS glue (SilgardTM Silicone Elastomer Kit). BSA (1% w/v) was added to the channel and incubated for at least 30 min at room temperature before the experiment. The channel was washed twice with RPMI incomplete media (pH 7.2, RPMI 1640, Gibco) and mounted on the stage of an inverted microscope (63× magnification, ZEISS, AxioObserver). Rosettes and single iRBCs were separated from the culture using magnetic purification and stained with CellBrite Red membrane dye (Biotium). These cells were adjusted to 10^6^ cells/mL in incomplete RPMI medium (pH 7.2). A total volume of 5-mL cell suspension was perfused at 0.05-Pa wall-shear stress, using the Harvard Apparatus syringe pump connected via silicon tubing with the flow chamber inlet. Measurements at high hematocrit were performed after the addition of packed HbA erythrocytes to the cell suspension and a total volume of 5 mL with 40% hematocrit was perfused at 0.05-Pa wall-shear stress into the flow chamber. The cell flow was recorded either using brightfield for low hematocrit or by recording the CellBrite signal with high frame rates (250–1000 fps) using a Zeiss inverted microscope (Axio Observer, 63× magnification, Ex/Em: 644/665, Cy5 filter set) and a high-speed camera (FastCam, Photron). During the experiment, the coordinates of the microscope stage were tracked and adjusted to correspond to the position of the recorded field within the chamber. Scanning through the height of the channel in 20-*μ*m steps allowed us to track the position of rosettes within the channel as well as along the length (12 mm) of the channel between inlets. Note that the channel length of 12 mm should be sufficient for margination measurements, as was shown in previous studies (41,42,43) for similar channel dimensions.

### Mesoscopic simulations

**Figure 2:**
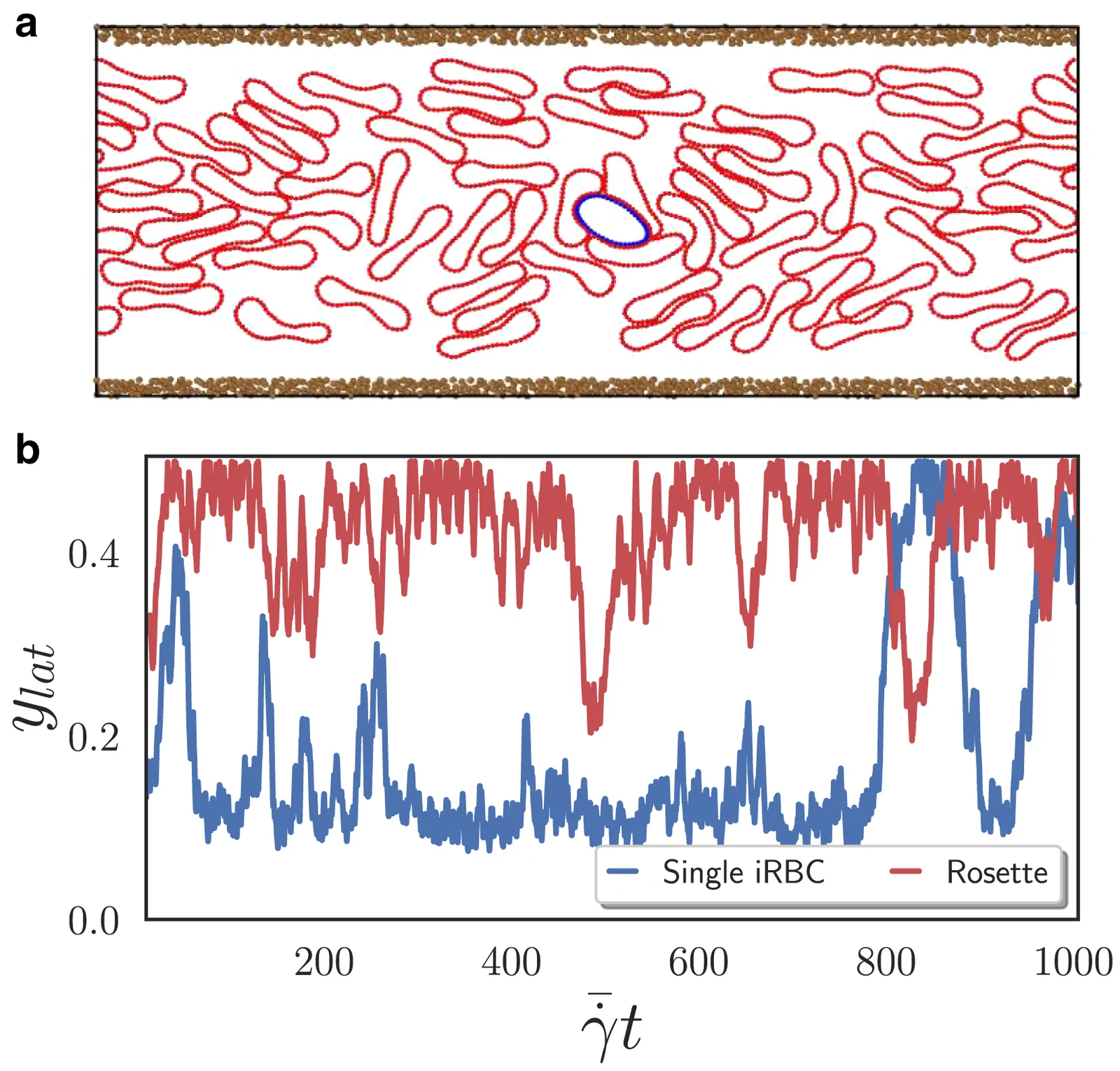
Time evolution of lateral position of an iRBC and rosette. (*a*) A snapshot of 2D simulations showing the formation of typical rosette structures. hRBCs are depicted in red and a single iRBC in blue, whereas brown DPD particles represent frozen wall particles. (*b*) Lateral position *y*_*lat*_ = (0.5 − |*y*_c_/*W*|) of rosette and a single iRBC in the channel as a function of time. For the case of rosettes, *k*_off_/*k*_on_ = 0.01 with *k*_on_ = 27.22/*τ*, whereas for a single iRBC, *k*_on_ = 0. In the simulations, hematocrit *H*_*t*_ = 40% and dimensionless shear rate ${\dot{\gamma }}^{*}=2.83$ are used.

We employ particle-based mesoscopic simulations in two dimensions (2D) for modeling RBCs and hydrodynamic interactions between them; see Fig. 2 *a*. Each RBC is represented as a 2D ring of *N*_*v*_ particles connected by *N*_*s*_ = *N*_*v*_ springs (39). The spring potential is given by

(Equation 1) $$V_{\text{sp}}=\sum_{i=1}^{N_{s}}\frac{k_{B}Tl_{m}\left(3x_{i}^{2}-2x_{i}^{3}\right)}{4l_{p}\left(1-x_{i}\right)}+\frac{k_{p}}{l_{i}}\text{,}$$

where the first term is an attractive worm-like chain potential, and the second term is a repulsive potential with the force coefficient *k*_p_. In the first term, $l_{p}$ is the persistence length, $l_{m}$ is the maximum spring extension, and $x_{i}=l_{i}/l_{m}$ with the spring length $l_{i}$.

The RBC membrane is also equipped with the bending energy

(Equation 2) $$V_{\text{bend}}=\sum_{i=1}^{N_{v}}k_{b}\left(1-\cos\left(\theta_{i}-\theta_{0}\right)\right)\text{,}$$

where *k*_b_ is the bending coefficient, *θ*_*i*_ is the instantaneous angle between two consecutive bonds at the vertex *i*, and *θ*_0_ is the preferred angle.

Finally, the RBC area is kept constant by applying the constraint potential

(Equation 3) $$V_{\text{area}}=\frac{k_{a}{\left(A-A_{0}\right)}^{2}}{2}\text{,}$$

where *k*_a_ is the area constraint coefficient, and *A* and *A*_0_ are the instantaneous and desired cell areas, respectively. The coefficient *k*_a_ is chosen large enough that the surface area of each RBC does not depart from *A*_0_ by more than 1% even under flow conditions.

Fluid flow is modeled by the dissipative particle dynamics (DPD) method (44,45), a particle-based mesoscopic hydrodynamics technique. DPD fluid consists of *N* particles that interact through three pairwise forces. The total force on particle *i* is given as a sum of forces from all its neighboring particles *j*(*i*) within the cutoff radius *r*_*c*_*:*

(Equation 4) $$F_{i}=\sum_{j\left(i\right)}F_{i,j}^{C}+F_{i,j}^{D}+F_{i,j}^{R}\text{,}$$

where $F_{i,j}^{C}$ is the conservative force, $F_{i,j}^{D}$ is the dissipative force, and $F_{i,j}^{R}$ is the random force. The conservative force controls fluid compressibility, the dissipative force accounts for fluid viscosity, and the random force controls thermal fluctuations. The pair of dissipative and random forces forms a thermostat that imposes a temperature *T* (45). DPD forces act between fluid particles, as well as between fluid and RBC membrane particles. Parameters of the DPD interaction forces are chosen such that no-slip boundary conditions are satisfied at the surfaces of RBCs (46). Fluids inside and outside RBCs have the same viscosity *η*.

The shape of hRBCs is biconcave, whereas an iRBC is represented by an ellipse, since the formation of rosettes starts from the early trophozoite stage, when the shape of iRBCs already deviates from the biconcave shape (7). Simulations are carried out in a rectangular channel with dimensions *L* = 19.2*D*_r_ and *W* = 7.2*D*_r_, where $D_{r}=2\sqrt{A_{0}/\pi }≃4.17\mu m$ is the effective hRBC diameter (*A*_0_ = 13.64 in simulations). The effective diameter of an iRBC is $D_{i}=2\sqrt{A_{i}/\pi }≃4.58\mu m$. The number of vertices for both hRBC and iRBC is set to *N*_*v*_ = 50. Initially, the iRBC is placed in the middle of the channel, whereas hRBCs are distributed homogeneously between the channel walls. Periodic boundary conditions are employed in the *x* direction, whereas in the *y* direction, the RBC suspension is bounded by two walls, which are modeled by frozen DPD particles. Flow rate *Q* through the channel is characterized by the dimensionless shear rate ${\dot{\gamma }}^{*}=\eta D_{r}^{3}\bar{\dot{\gamma }}/\kappa$, where $\kappa =k_{b}l_{0}$ is the bending rigidity with an equilibrium spring length $l_{0}=0.092D_{r}$, *η* is the dynamic fluid viscosity (*η* = 72.16 in simulations), and $\bar{\dot{\gamma }}=Q/W^{2}$ is the average (or pseudo) shear rate. Note that $\tau =\eta D_{r}^{3}/\kappa$ is a characteristic relaxation time of a hRBC, which will be used as a timescale. The bending coefficient *k*_*b*_ for healthy and infected RBCs is set to 50*k*_B_*T* and 500*k*_B_*T* (*k*_B_*T* = 1 in simulations), respectively. The preferred angle *θ*_0_ is set to zero for both hRBCs and iRBCs. The Young’s modulus $Y=\left(-\partial^{2}V_{\text{sp}}/\partial l^{2}\right){|}_{l_{0}}$ of both hRBCs and iRBCs is characterized by $\alpha =YD_{r}^{2}/\kappa =1340$ (39). For comparison, in physical units, we can assume *D*_r_ = 4.17*μ*m, *κ*_*r*_ = 50*k*_B_*T*, and *η* = 10^−3^Pa·s. This yields a characteristic RBC relaxation time, $\tau =\eta D_{r}^{3}/\kappa_{r}≃0.3528s$. By comparing this relaxation time with that in model units, we find that the average shear rate in our simulations corresponds to a range from 2.68s^−1^ to 10.72s^−1^ in physical units. For *W* = 30*μ*m, the corresponding flow rates *Q* range from 2412*μ*m^2^/s to 9648*μ*m^2^/s.

Adhesion interactions between hRBCs and iRBCs are implemented through dynamic bond formation and dissociation with predefined on- and off-rates. Membrane vertices on the surface of RBCs serve as receptors and ligands for bond formation. When the distance between receptor and ligand is less than a critical distance of 0.12*D*_r_, a bond is created with constant on-rate *k*_on_. Existing bonds can break with a force-dependent off-rate $k_{\text{off}}\left(f\right)=k_{\text{off}}^{0}\;\exp\;\left(f/f_{D}\right)$, according to the Bell’s model (47). Note that there are no adhesive interactions between different hRBCs.

## Results

We investigated rosette margination in blood flow by DPD simulations for various parameters, such as flow rate, hematocrit, and binding kinetic rates between hRBCs and iRBCs. To quantify margination strength, we measure the (normalized) lateral position *y*_*lat*_=(0.5 − |*y*_c_/*W*|) of the iRBC center of mass for both single iRBCs and rosettes. Since the iRBC typically resides close to the geometric center of a rosette, usage of the iRBC center of mass to define *y*_lat_ for rosettes is appropriate. When the iRBC is close to one of the walls (strong margination), *y*_*lat*_ is close to zero, whereas *y*_*lat*_ ≈ 0.5, when the iRBC is near the channel center. The binding kinetic rates between the iRBC and hRBCs are set to represent different adhesion strengths: 1) a stable rosette with *k*_off_/*k*_on_ = 0.01 such that no dissociation of formed RBC clusters occurs in blood flow; 2) a dynamic rosette with *k*_off_/*k*_on_ = 5 such that hRBCs within the cluster can dynamically be replaced by new hRBCs; and 3) a single iRBC with *k*_off_/*k*_on_ = *∞* such that *k*_on_ = 0 and no bonds can form. In all simulations with bond formation (i.e., *k*_on_ > 0), the on-rate *k*_on_ = 27.22/*τ* remains fixed. Each simulation is run for approximately 3 × 10^7^ timesteps, corresponding to a total time of 220.4*τ* (or about 78 s). The lateral position is sampled every 10^4^ timesteps or every 0.073*τ* in time.

### Clustering reduces margination

Fig. 2 *a* shows a representative snapshot of a rosette formed by one iRBC and three hRBCs in blood flow at *H*_*t*_ = 40% and ${\dot{\gamma }}^{*}=2.83$. The rosette moves primarily near the center of the channel, as indicated in Fig. 2 *b* by its lateral position *y*_*lat*_. In contrast, a single iRBC remains mainly near the walls with *y*_*lat*_ ≈ 0.1. Therefore, single iRBCs exhibit pronounced migration toward the walls, whereas the margination of rosettes appears to be poor. Single iRBCs marginate well due to their nearly nondeformable ellipse shape that is close to circular. Our previous studies have shown that rigid circular or ellipse-like particles marginate well in blood flow, whereas particle deformabilty can strongly reduce margination (32,48). In comparison, rosette structures consist of a stiff iRBC covered by several deformable hRBCs. As a result, the formed cluster is much more deformable than a single stiff iRBC. The high deformability of rosettes results in large lift forces due to hydrodynamic interactions with the walls, which push rosettes away from the walls (33,49). In addition to the deformability of rosette structures, they are significantly larger in size than single iRBCs. The lift force on a deformable particle near a wall is proportional to the particle size to the power of 3–4 (34,50), indicating that the force driving rosettes away from the walls is much larger than that for single iRBCs.

**Figure 3:**
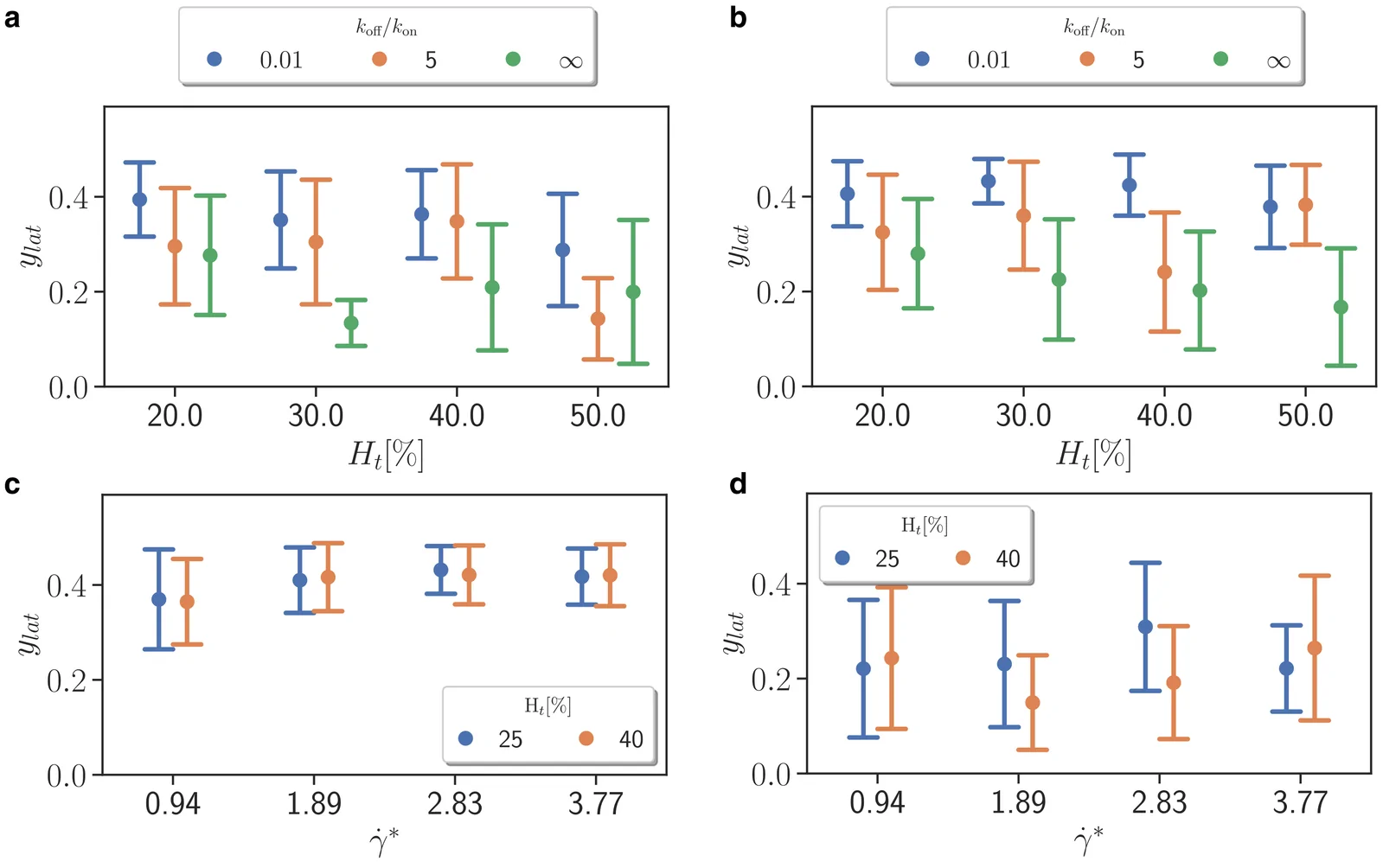
Average lateral position *y*_*lat*_ of rosettes (*blue, orange*) and single iRBCs (*green*) for different hematocrit values and flow rates. ${\bar{y}}_{lat}$ and its standard deviation for (*a*) ${\dot{\gamma }}^{*}=0.94$ and (*b*) ${\dot{\gamma }}^{*}=2.83$ as a function of hematocrit *H*_*t*_. *k*_off_/*k*_on_ = 0.01 and *k*_off_/*k*_on_ = 5 represent the cases of stable and dynamic rosettes, whereas *k*_off_/*k*_on_ = *∞* (i.e., *k*_on_ = 0) corresponds to single iRBCs, since rosettes do not form in these simulations. ${\bar{y}}_{lat}$ and its standard deviation for (*c*) *k*_off_/*k*_on_ = 0.01 and (*d*) *k*_off_/*k*_on_ = *∞* as a function of flow rate for two different *H*_*t*_ values.

Fig. 3 *a* shows average lateral position ${\bar{y}}_{lat}$ of rosettes and single iRBCs as a function of *H*_*t*_ for a low flow rate with ${\dot{\gamma }}^{*}=0.94$. The three values of *k*_off_/*k*_on_ = 0.01, 5, and *∞* represent the cases of stable and dynamic rosettes and single iRBCs, respectively. For single iRBCs, margination increases with increasing *H*_*t*_, indicated by a decreasing ${\bar{y}}_{lat}$. For the two cases involving rosettes, margination appears to improve only beyond a hematocrit of *H*_*t*_ = 40%. A possible explanation for the enhanced margination at higher hematocrit is that the size of the rosettes becomes comparable to the thickness of the RBC-free layer between the channel walls and the RBC-rich core. It is also important to note that the cell-free layer becomes thinner at this flow rate, making it more challenging to clearly define margination. It has been shown that deformable particles may also have good margination when they fit well within the RBC-free layer in blood flow (48). For larger hematocrits, *H*_*t*_ > 30%, the thickness of the RBC-free layer decreases, and the margination of rosettes becomes weaker. Fig. 3 *a* also shows that loose dynamic rosettes have slightly better margination properties in comparison to the stable rosettes. As expected, margination for both rosette cases is generally worse than that for single iRBCs. Fig. 3 *b* shows the average lateral position ${\bar{y}}_{lat}$ of rosettes and single iRBCs as a function of *H*_*t*_ at ${\dot{\gamma }}^{*}=2.83$. For this flow rate, the dependence of margination on *H*_*t*_ appears to be weak, such that ${\bar{y}}_{lat}$ is nearly independent of hematocrit. Similar to the case of a low flow rate of ${\dot{\gamma }}^{*}=0.94$ in Fig. 3 *a*, margination at a high flow rate of ${\dot{\gamma }}^{*}=2.83$ is best for single iRBCs, worst for stable rosettes, and falls in between these two extreme cases for dynamic rosettes. At both flow rates, the error bars corresponding to dynamic rosettes or *k*_off_/*k*_on_ = 5 are slightly larger. This is because repeated binding and unbinding of hRBCs to iRBCs leads to a variable coordination number and fluctuating rosette size, resulting in significant variations in the lateral position. Furthermore, even for single iRBCs and stable rosettes, there exist occasional excursions between the channel center and the wall, which also leads to an increase in the standard deviation of ${\bar{y}}_{lat}$.

Fig. 3 *c* presents ${\bar{y}}_{lat}$ as a function of nondimensional shear rate ${\dot{\gamma }}^{*}$ for the case of stable rosettes with *k*_off_/*k*_on_ = 0.01. ${\bar{y}}_{lat}≃0.4$ indicates poor margination, which is nearly independent of ${\dot{\gamma }}^{*}$. This is likely due to the fact that a stable rosette keeps its constituent cells within the cluster, regardless of shear stresses exerted by the flow. Furthermore, there is nearly no difference in margination of stable rosettes for the two hematocrits of 25% and 40%. Fig. 3 *d* shows ${\bar{y}}_{lat}$ as a function of ${\dot{\gamma }}^{*}$ for the case of single iRBCs (i.e., *k*_off_/*k*_on_ = *∞*). iRBCs exhibit pronounced margination, which slightly reduces with increasing flow rate. A reduction in margination for deformable particles at high flow rates is due to their deformability, because it leads to an increase in the lift force that drives them away from the walls (39,40,48). Furthermore, margination of iRBCs is slightly better for *H*_*t*_ = 40% compared with *H*_*t*_ = 25%, consistent with earlier simulation results for WBCs (39,40).

Previous work on margination of WBCs (27,28,29) and drug delivery carriers (30,31,32), and the results for rosette margination presented here strongly suggest that suspended particles in blood flow either marginate well or not. As a result, there is always a preferred position either near a wall or near the center, depending on particle properties and flow conditions. Thus, when particle position is close to the preferred position, the suspended particle may not show much migration, but if the particle position significantly differs from the preferred position, it will gradually migrate toward the preferred position. Note that the margination process does not exhibit asymptotic behavior toward a specific lateral position, and transitions between the channel center and the wall can always occur. The frequency of these transitions is indeed influenced by several factors, including flow rate, hematocrit, and the properties of suspended particles. This also suggests that margination results should decorrelate from the initial position of the iRBC, when several transitions between the channel center and the wall are observed during the length of simulations. The trajectories in Fig. 2 *b* show several of such transitions, confirming that the length of simulations is long enough to reach steady state and to decorrelate margination results from the initial position of iRBCs in the channel.

### Adhesion strength affects rosette margination

**Figure 4:**
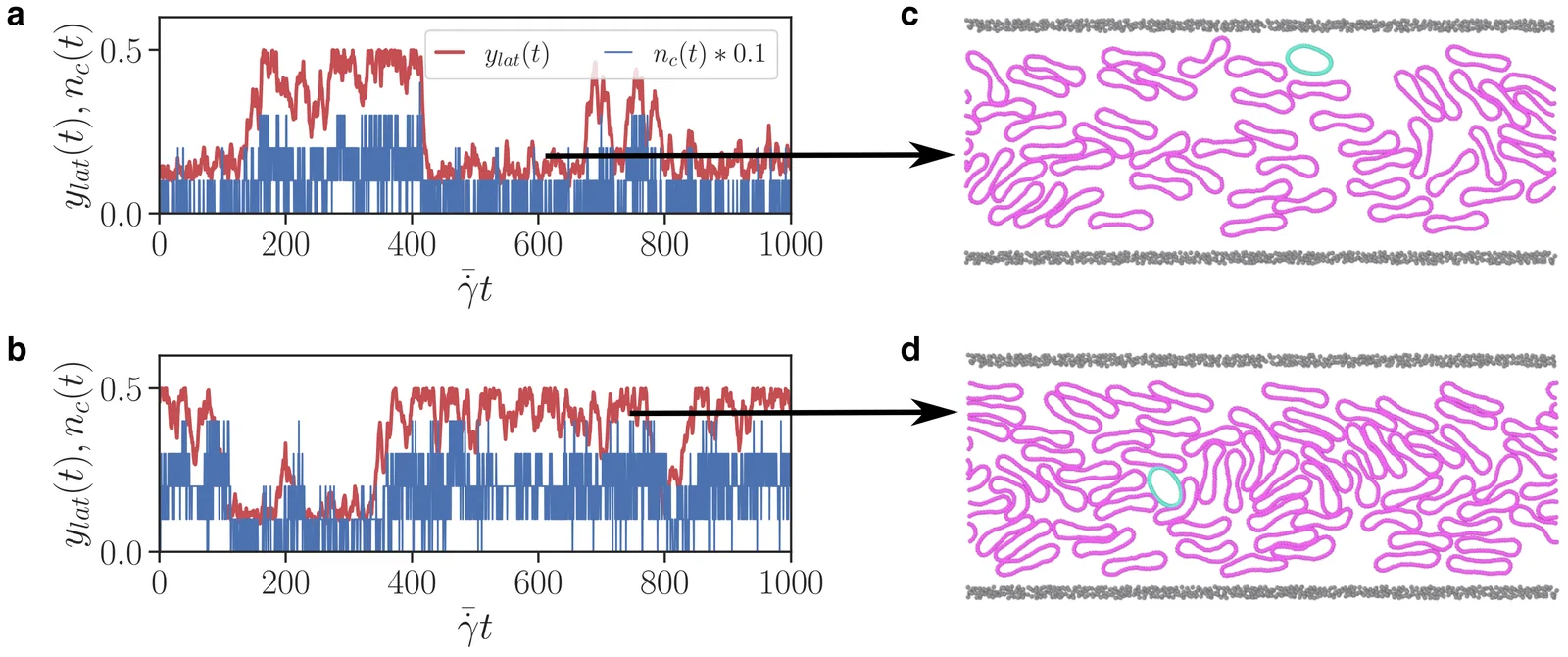
Correlation of rosette margination with the coordination number *n*_*c*_(*t*) (i.e., the number of hRBCs bound to iRBC). Comparison of *y*_*lat*_(*t*) and *n*_*c*_(*t*) for (a) *H*_*t*_ = 30% at ${\dot{\gamma }}^{*}=1.89$ and *k*_off_/*k*_on_ = 5, and (*b*) *H*_*t*_ = 40% at ${\dot{\gamma }}^{*}=2.83$ and *k*_off_/*k*_on_ = 3. The coordination number is multiplied by a factor of 0.1 for a better comparison with *y*_*lat*_(*t*). The smaller the coordination number is, the better the rosette margination is. (*c* and *d*) Snapshots of simulations at the specified times points, indicated by the black arrows. See also Videos S2 and S3.

To corroborate the conclusion that single iRBCs marginate significantly better than stable rosettes consisting of several cells, we study the relation between rosette lateral position *y*_*lat*_ and coordination number *n*_*c*_ or the number of hRBCs attached to the iRBC. *n*_*c*_ is computed by counting the number of hRBCs that are bound by at least one bond to the iRBC within a rosette. Fig. 4 *a* and *b* presents *y*_*lat*_(*t*) and *n*_*c*_(*t*) as a function of time for two different flow and adhesion conditions, where the ratio *k*_off_/*k*_on_ is selected such that dynamic rosettes with a different number of adhered hRBCs are expected to be observed, depending on local flow stresses. The coordination number is correlated well with the lateral rosette position. Therefore, adhesion strength clearly affects rosette margination, since it governs its size and deformability. It is worthwhile to mention that for moderate adhesion strengths shown in Fig. 4, hRBC-to-iRBC binding events are continuously occurring, making rosettes very dynamic. The case of dynamic bonds interconnects well the two limiting cases of no bindings and permanent bindings, which exhibit pronounced and poor margination, respectively. Fig. 4 *c* and *d* also illustrates typical simulation snapshots, where a cluster formed by the iRBC and seveal hRBCs is near the channel center, whereas a single iRBC is close to the wall (see also Videos S2 and S3).

Video S2. Blood simulation with a single iRBC for *H*_*t*_ = 30%, $\dot{\gamma }$∗ = 1.89, and *k*_off_/*k*_on_ = 5 A rosette is formed by the iRBC and several hRBCs and is located near the channel center. See also the snapshot in Fig. 4 *c*.

Video S3. Simulated iRBC in blood flow for *H*_*t*_ = 40%, $\dot{\gamma }$∗ = 2.83, and *k*_off_/*k*_on_ = 3 No stable rosette structures are formed, and the iRBC flows primarily near the wall.

### Size and deformation of rosettes in flow

**Figure 5:**
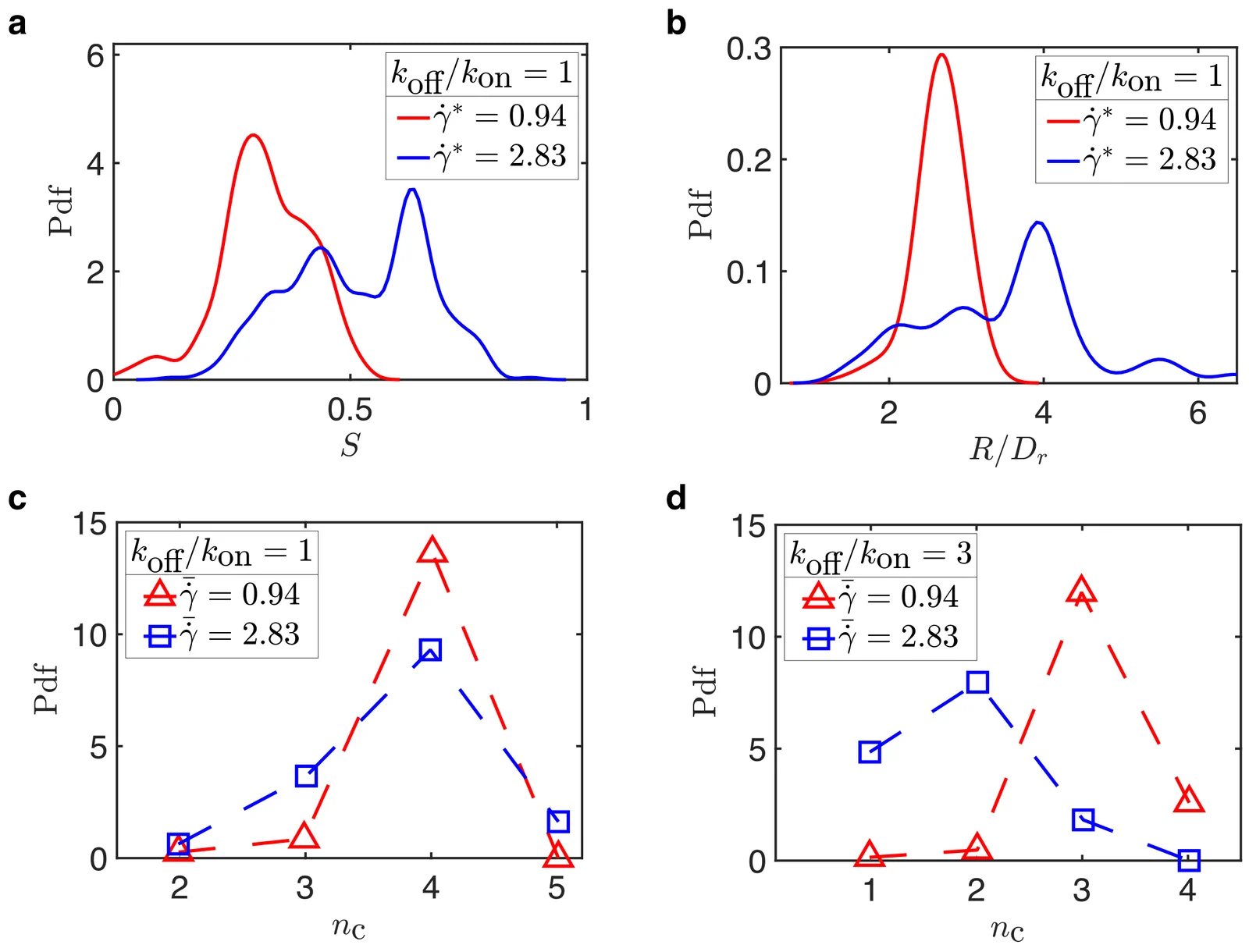
Probability density functions of shape, size, and coordination numbers of a rosette. Probability density functions (Pdf) of (*a*) the shape asymmetry S and (*b*) the rosette size R for two different values of flow rates at a fixed *k*_off_/*k*_on_ = 1 and *H*_*t*_ = 30%. Distributions of the coordination number *n*_*c*_ for (*c*) *k*_off_/*k*_on_ = 1 and (*d*) *k*_off_/*k*_on_ = 3, using two different values of ${\dot{\gamma }}^{*}$ at *H*_*t*_ = 30%.

To quantify rosette size and deformability, we also employ the gyration tensor computed from all RBC particles forming the rosette. The effective rosette size R and its shape asymmetry S are defined as

(Equation 5) $$R=\sqrt{\Lambda_{\max}^{2}+\Lambda_{\min}^{2}},S=\frac{\Lambda_{\max}-\Lambda_{\min}}{\Lambda_{\max}+\Lambda_{\min}}\text{,}$$

where Λ_*max*_ and Λ_*min*_ are the maximum and minimum eigenvalues of the gyration tensor, respectively. Here, *S* = 0 indicates circular and *S* = 1 strongly elongated aggregates. Fig. 5 *a* and *b* shows distributions of S and R for two distinct values of ${\dot{\gamma }}^{*}$ at a fixed *k*_off_/*k*_on_ = 1 and *H*_*t*_ = 30%. The shape asymmetry S is clearly larger for ${\dot{\gamma }}^{*}=2.83$ in comparison to ${\dot{\gamma }}^{*}=0.94$, indicating a strong elongation of rosettes at large flow rates. Due to the significant deformation of rosettes at large ${\dot{\gamma }}^{*}$, the effective size R of rosettes is also considerably larger at ${\dot{\gamma }}^{*}=2.83$ than at ${\dot{\gamma }}^{*}=0.94$.

Fig. 5 *c* and *d* also shows distributions of the coordination number *n*_*c*_ for two different values of *k*_off_/*k*_on_. In the case of *k*_off_/*k*_on_ = 1, differences in the *n*_*c*_ distributions for different flow rates are small, indicating that rosette clusters are stable within this range of ${\dot{\gamma }}^{*}$, with the most probable number of four adhered hRBCs within the rosette cluster. However, in the case of *k*_off_/*k*_on_ = 3, rosettes at ${\dot{\gamma }}^{*}=2.83$ are smaller than at ${\dot{\gamma }}^{*}=0.94$, because they are dynamic and can be broken by high enough shear stresses in blood flow. Furthermore, there is a frequent exchange of hRBCs for the dynamic rosettes at *k*_off_/*k*_on_ = 3, compared with the stable rosettes at *k*_off_/*k*_on_ = 1.

### Experimentally observed rosette margination

**Figure 6:**
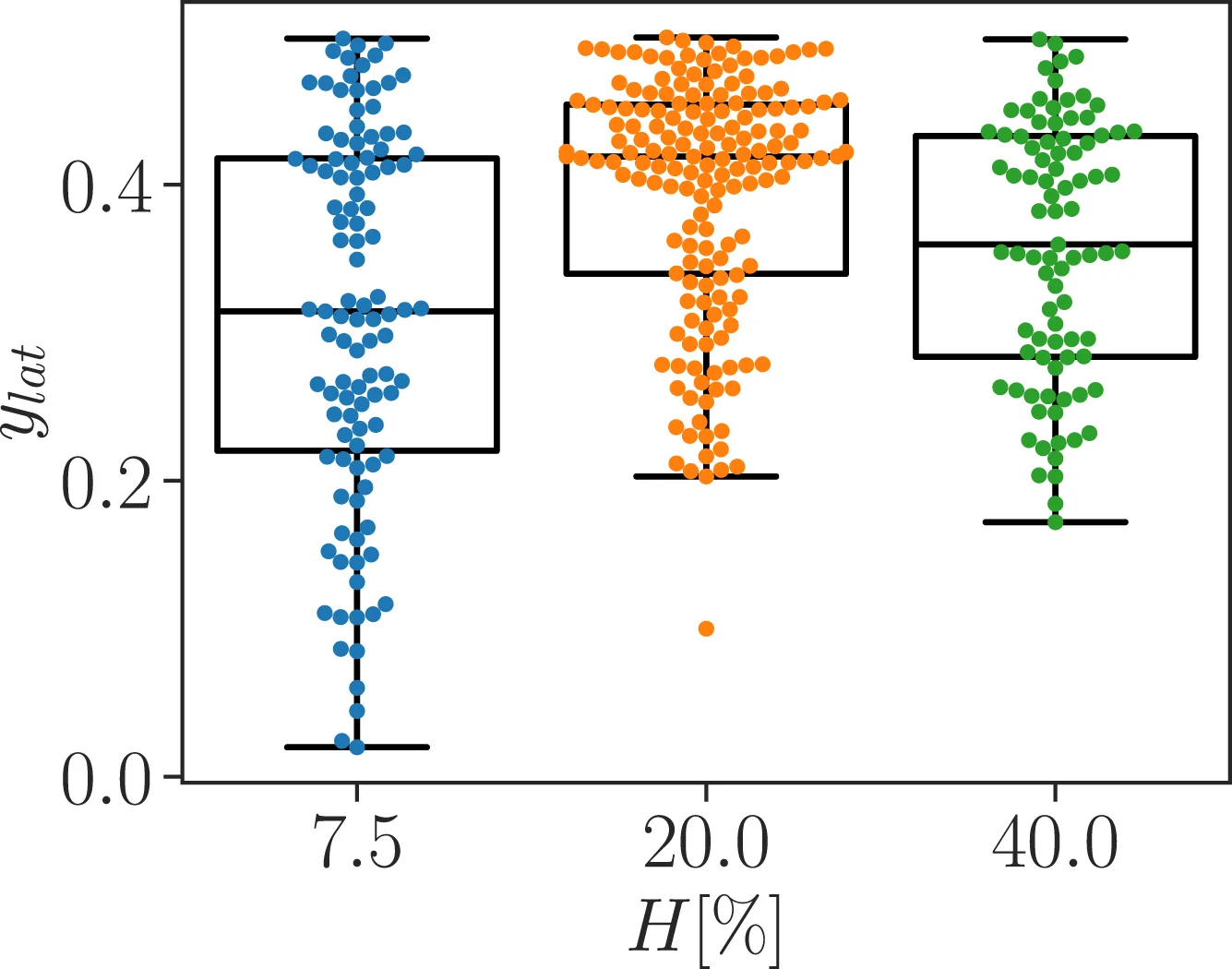
Rosette lateral position *y*_*lat*_ within the channel for three different hematocrit values from microfluidic experiments. Here, lateral position is defined as *y*_*lat*_ = 0.5 − (|*z|*/*W*), where *z* represents the *z*-component of the rosette’s position in microfluidic chamber. For each hematocrit, the sample size is approximately 100.

To experimentally corroborate the poor margination of rosette clusters in blood flow, we have performed flow chamber microfluidic experiments (see Video S4). The experimental microfluidic setup is depicted in Fig. 1 *b*. Similar to computer simulations, the experiments were performed for three different hematocrits, including *H*_*t*_ = 7.5%, *H*_*t*_ = 20%, and *H*_*t*_ = 40%. Fig. 6 shows the lateral position *y*_*lat*_ of rosettes for the three hematocrit values, indicating that the majority of them are located near the channel center. The average values of *y*_*lat*_ ≈ 0.3–0.4 are similar to those obtained in simulations of stable rosettes with *k*_off_/*k*_on_ = 0.01 (see Fig. 3). In experiments, rosettes have been observed to rotate in flow, a phenomenon not seen in simulations since rosettes are primarily located in the channel center. This discrepancy may also arise because the simulations are conducted in two dimensions. The experiments were performed at a flow rate characterized by the wall shear stress of 0.05 Pa, which corresponds to ${\dot{\gamma }}^{*}≃8.2$ and is comparable to the range of values explored in the simulations. Larger flow rates lead to difficulties in rosette tracking. Note that, the performed simulations suggest that no improvement in the margination of stable rosettes should be expected when the flow rate is increased, because larger shear stresses lead to stronger rosette deformations. In conclusion, microfluidic experiments of blood flow in a channel confirm the poor margination of rosettes predicted by the simulations.

Video S4. Flow chamber microfluidic experiment at the wall shear stress of 0.05 Pa Several rosette structures are observed, as they are transported by the flow.

## Discussion

Rosettes have been linked to severe malaria and have been suggested to play a key role in the disease progression (11,12,13,51). To better understand the behavior of rosette clusters in blood flow, we have performed numerical simulations and microfluidic experiments, which quantify the margination propensity of rosettes in blood flow. A high propensity for the migration toward a vessel wall (i.e., efficient margination) can facilitate the adhesion of iRBCs and rosettes to vascular endothelium and, thus, allow the parasites to avoid passage through the spleen, where infected RBCs are sorted out. On the contrary, poor margination of rosettes would substantially reduce their adhesion to the vascular endothelium.

**Figure 7:**
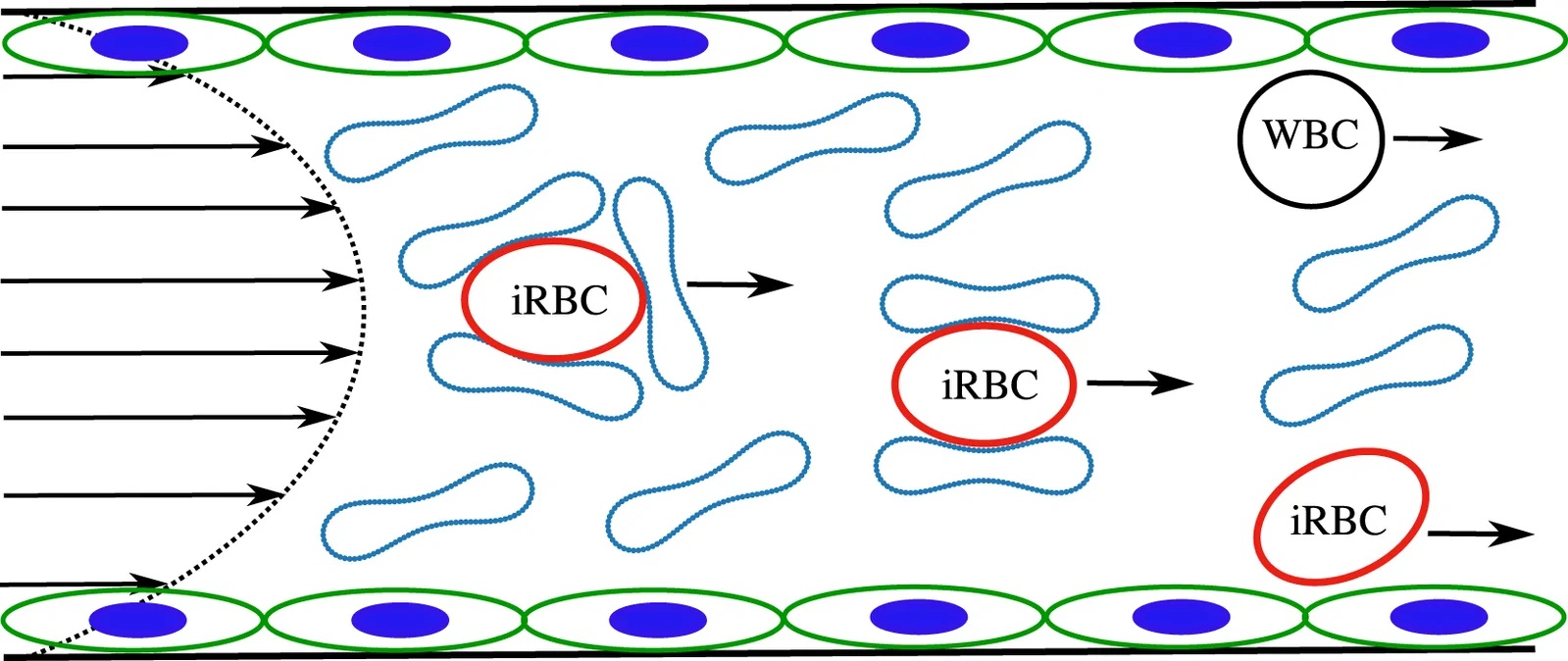
A sketch summarizing rosette margination in blood flow. Single iRBCs marginate well, whereas the margination of rosettes worsens with increasing their size, which is indicated by the lateral rosette positions. For comparison, an efficient margination of nearly spherical white blood cells (WBCs) is also shown. The green cells are endothelial cells with a nucleus shown in blue color.

Fig. 7 summarizes the margination of rosettes in blood flow obtained from simulations and experiments. Stable rosettes, which consist of one iRBC and several permanently bound hRBCs, have poor margination properties. Thus, they are expected to reside primarily near the center of a vessel and have a limited ability to adhere to the endothelium. The poor margination of stable rosettes is due to their deformability and large size as a cell cluster. When the adhesion between iRBCs and hRBCs is weak enough to allow a continuous exchange of adhered hRBCs, dynamic rosette structures are formed, which marginate better than stable rosettes. The more efficient margination of dynamic rosettes is mainly due to a reduction in their size (i.e., smaller number of adhered hRBCs), since the hydrodynamic lift force that drives deformable particles away from the walls scales as the particle size to the power of 3–4 (34,50,52). Nevertheless, the most efficient margination is found for single iRBCs or when the adhesive interactions between iRBCs and hRBCs can be neglected with respect to shear stresses exerted by blood flow. This is mainly due to the low deformability of iRBCs, whose margination is comparable with the margination of WBCs in blood flow (38,39,40). Note that the margination of WBCs can be drastically reduced when they become deformable (39,40). However, some quantitative differences between 2D and 3D simulations do arise. Notably, margination in 2D tends to occur at higher hematocrit levels compared with 3D; for instance, 2D simulations often require a hematocrit increase of approximately 0.1–0.2 to reproduce the same RBC-free layer thickness observed in 3D cylindrical geometries (53). This discrepancy likely stems from geometric differences, particularly the reduced number of interacting neighbors in 2D versus 3D, which influences both cell packing and lateral migration dynamics. Furthermore, in 3D, more hRBCs can be packed around an iRBC. However, due to the discocyte shape of RBCs, packing also gets geometrically and elastically more complicated, resulting in a variety of aggregate shapes (compare the emergent structure of RBC doublets (54)).

We also want to briefly comment on the role of cell adhesion—both the aggregation between hRBCs and the cytoadhesion of iRBCs to the vessel walls. Both effects have been neglected or suppressed in our simulations and experiments. Aggregation of hRBCs may play a role in margination at high hematocrit in the venular part of the microvasculature due to relatively low shear rates (39,40). For cytoadhesion, margination is a necessary prerequisite, so that our results of reduced rosette margination imply also a partial suppression of cytoadhesion.

Blood flow in the microvasculature is much more complex than the flow in a straight channel, due to the intricate tree-like geometry of microvascular networks with numerous bifurcations and vessel junctions. Localization of rosettes near the vessel center may enhance their collision with the endothelium at bifurcations due to the separation of flow following two daughter vessels. For instance, perfusion experiments (14) with rosette-positive and rosette-negative cell lines have shown an increased aggregation of RBCs near venular junctions for the rosette-positive line in comparison to the rosette-negative cell line. This suggests that rosette formation has evolved as a strategy to deliver iRBCs to special places in the microvasculature. The formation of RBC aggregates near venular junctions may eventually lead to the local blockage of blood flow. Furthermore, rosette structures should result in an increased risk of the blockage of small vessels, since single iRBCs are already able to clog small capillaries (55). The increased risk of vessel blockage for rosette-positive parasite strains is consistent with the fact that the formation of rosettes is often associated with severe malaria (11,13,51).

## Data and code availability

Data of this study are available from the corresponding author upon reasonable request.

## Acknowledgments

We acknowledge computing time on the supercomputer JURECA (56) at Forschungszentrum Jülich under grant no. actsys. This work was supported by the 10.13039/501100001659Deutsche Forschungsgemeinschaft (DFG, German Research Foundation) Projektnummer 240245660-SFB 1129, project 4 (U.S.S.; M.L.). G.G. and U.S.S. wish to express their gratitude to Erich Sackmann for many inspiring discussions on the physics of cells.

## Author contributions

M.L., U.S.S., G.G., and D.A.F. designed the research project. A.K.D. performed the simulations and analyzed the obtained data. M.P. performed experiments and analyzed the data. All authors participated in the discussions and writing of the manuscript.

## Declaration of interests

The authors declare no competing interests.

## References

[1] Miller L.H., Baruch D.I., Doumbo O.K. (2002). The pathogenic basis of malaria. Nature.

[2] White N.J., Pukrittayakamee S., Dondorp A.M. (2014). Malaria. Lancet.

[3] Quadt K.A., Barfod L., Hviid L. (2012). The Density of Knobs on Plasmodium falciparum-Infected Erythrocytes Depends on Developmental Age and Varies among Isolates. PLoS One.

[4] Gruenberg J., Allred D.R., Sherman I.W. (1983). Scanning electron microscope-analysis of the protrusions (knobs) present on the surface of Plasmodium falciparum-infected erythrocytes. J. Cell Biol..

[5] Nagao E., Kaneko O., Dvorak J.A. (2000). Plasmodium falciparum-Infected Erythrocytes: Qualitative and Quantitative Analyses of Parasite-Induced Knobs by Atomic Force Microscopy. J. Struct. Biol..

[6] Brown H., Hien T.T., Turner G. (1999). Evidence of blood-brain barrier dysfunction in human cerebral malaria. Neuropathol. Appl. Neurobiol..

[7] Waldecker M., Dasanna A.K., Lanzer M. (2017). Differential time-dependent volumetric and surface area changes and delayed induction of new permeation pathways in P. falciparum-infected hemoglobinopathic erythrocytes. Cell. Microbiol..

[8] Esposito A., Choimet J.-B., Tiffert T. (2010). Quantitative imaging of human red blood cells infected with Plasmodium falciparum. Biophys. J..

[9] Li J., Dao M., Suresh S. (2005). Spectrin-level modeling of the cytoskeleton and optical tweezers stretching of the erythrocyte. Biophys. J..

[10] Abkarian M., Massiera G., Braun-Breton C. (2011). A novel mechanism for egress of malarial parasites from red blood cells. Blood.

[11] Carlson J., Helmby H., Wahlgren M. (1990). Human cerebral malaria: association with erythrocyte rosetting and lack of anti-rosetting antibodies. Lancet.

[12] Treutiger C.-J., Hedlund I., Wahlgren M. (1992). Rosette formation in Plasmodium falciparum isolates and anti-rosette activity of sera from Gambians with cerebral or uncomplicated malaria. Am. J. Trop. Med. Hyg..

[13] Rowe A., Obeiro J., Marsh K. (1995). Plasmodium falciparum rosetting is associated with malaria severity in Kenya. Infect. Immun..

[14] Kaul D.K., Roth E.F., Handunnetti S.M. (1991). Rosetting of Plasmodium falciparum-infected red blood cells with uninfected red blood cells enhances microvascular obstruction under flow conditions. Blood.

[15] Moll K., Palmkvist M., Wahlgren M. (2015). Evasion of immunity to Plasmodium falciparum: rosettes of blood group A impair recognition of PfEMP1. PLoS One.

[16] Chotivanich K.T., Dondorp A.M., Udomsangpetch R. (2000). The resistance to physiological shear stresses of the erythrocytic rosettes formed by cells infected with Plasmodium falciparum. Ann. Trop. Med. Parasitol..

[17] Goel S., Palmkvist M., Wahlgren M. (2015). RIFINs are adhesins implicated in severe Plasmodium falciparum malaria. Nat. Med..

[18] Nunes-Alves C. (2015). RIFINs promote rosette formation during malaria. Nat. Rev. Microbiol..

[19] Rowe J.A., Claessens A., Arman M. (2009). Adhesion of Plasmodium falciparum-infected erythrocytes to human cells: molecular mechanisms and therapeutic implications. Expert Rev. Mol. Med..

[20] Lim Y.B., Thingna J., Lim C.T. (2017). Single molecule and multiple bond characterization of catch bond associated cytoadhesion in malaria. Sci. Rep..

[21] Rieger H., Yoshikawa H.Y., Lanzer M. (2015). Cytoadhesion of Plasmodium falciparum–infected erythrocytes to chondroitin-4-sulfate is cooperative and shear enhanced. Blood.

[22] Fedosov D.A., Caswell B., Karniadakis G.E. (2011). Wall shear stress-based model for adhesive dynamics of red blood cells in malaria. Biophys. J..

[23] Dasanna A.K., Fedosov D.A., Schwarz U.S. (2019). State diagram for wall adhesion of red blood cells in shear flow: from crawling to flipping. Soft Matter.

[24] Dasanna A.K., Lansche C., Schwarz U.S. (2017). Rolling Adhesion of Schizont Stage Malaria-Infected Red Blood Cells in Shear Flow. Biophys. J..

[25] Ishida S., Ami A., Imai Y. (2017). Factors diminishing cytoadhesion of red blood cells infected by plasmodium falciparum in arterioles. Biophys. J..

[26] Lansche C., Dasanna A.K., Lanzer M. (2018). The sickle cell trait affects contact dynamics and endothelial cell activation in Plasmodium falciparum-infected erythrocytes. Commun. Biol..

[27] Bagge U., Karlsson R. (1980). Maintenance of white blood cell margination at the passage through small venular junctions. Microvasc. Res..

[28] Firrell J.C., Lipowsky H.H. (1989). Leukocyte margination and deformation in mesenteric venules of rat. Am. J. Physiol..

[29] Fedosov D.A., Noguchi H., Gompper G. (2014). Multiscale modeling of blood flow: from single cells to blood rheology. Biomech. Model. Mechanobiol..

[30] Decuzzi P., Lee S., Ferrari M. (2005). A theoretical model for the margination of particles within blood vessels. Ann. Biomed. Eng..

[31] Lee T.-R., Choi M., Decuzzi P. (2013). On the near-wall accumulation of injectable particles in the microcirculation: smaller is not better. Sci. Rep..

[32] Müller K., Fedosov D.A., Gompper G. (2014). Margination of micro- and nano-particles in blood flow and its effect on drug delivery. Sci. Rep..

[33] Cantat I., Misbah C. (1999). Lift force and dynamical unbinding of adhering vesicles under shear flow. Phys. Rev. Lett..

[34] Abkarian M., Lartigue C., Viallat A. (2002). Tank treading and unbinding of deformable vesicles in shear flow: determination of the lift force. Phys. Rev. Lett..

[35] Imai Y., Nakaaki K., Yamaguchi T. (2011). Margination of red blood cells infected by Plasmodium falciparum in a microvessel. J. Biomech..

[36] Chen Y., Li D., Chen H. (2017). Margination of stiffened red blood cells regulated by vessel geometry. Sci. Rep..

[37] Hou H.W., Bhagat A.A.S., Lim C.T. (2010). Deformability based cell margination—a simple microfluidic design for malaria-infected erythrocyte separation. Lab Chip.

[38] Freund J.B. (2007). Leukocyte margination in a model microvessel. Phys. Fluids.

[39] Fedosov D.A., Fornleitner J., Gompper G. (2012). Margination of white blood cells in microcapillary flow. Phys. Rev. Lett..

[40] Fedosov D.A., Gompper G. (2014). White blood cell margination in microcirculation. Soft Matter.

[41] Fitzgibbon S., Spann A.P., Shaqfeh E.S.G. (2015). In vitro measurement of particle margination in the microchannel flow: effect of varying hematocrit. Biophys. J..

[42] Katanov D., Gompper G., Fedosov D.A. (2015). Microvascular blood flow resistance: role of red blood cell migration and dispersion. Microvasc. Res..

[43] Mehrabadi M., Ku D.N., Aidun C.K. (2016). Effects of shear rate, confinement, and particle parameters on margination in blood flow. Phys. Rev. E.

[44] Hoogerbrugge P.J., Koelman J.M.V.A. (1992). Simulating microscopic hydrodynamic phenomena with dissipative particle dynamics. EPL (Europhysics Letters).

[45] Español P., Warren P. (1995). Statistical mechanics of dissipative particle dynamics. Europhys. Lett..

[46] Fedosov D.A., Caswell B., Karniadakis G.E. (2010). A multiscale red blood cell model with accurate mechanics, rheology, and dynamics. Biophys. J..

[47] Bell G.I. (1978). Models for the specific adhesion of cells to cells: a theoretical framework for adhesion mediated by reversible bonds between cell surface molecules. Science.

[48] Müller K., Fedosov D.A., Gompper G. (2016). Understanding particle margination in blood flow - a step toward optimized drug delivery systems. Med. Eng. Phys..

[49] Meßlinger S., Schmidt B., Gompper G. (2009). Dynamical regimes and hydrodynamic lift of viscous vesicles under shear. Phys. Rev. E.

[50] Callens N., Minetti C., Podgorski T. (2008). Hydrodynamic lift of vesicles under shear flow in microgravity. Europhys. Lett..

[51] Dondorp A.M., Pongponratn E., White N.J. (2004). Reduced microcirculatory flow in severe falciparum malaria: pathophysiology and electron-microscopic pathology. Acta Trop..

[52] Podgorski T., Callens N., Misbah C. (2011). Dynamics of vesicle suspensions in shear flow between walls. Microgravity Sci. Technol..

[53] Rack K., Huck V., Gompper G. (2017). Margination and stretching of von Willebrand factor in the blood stream enable adhesion. Sci. Rep..

[54] Hoore M., Yaya F., Fedosov D.A. (2018). Effect of spectrin network elasticity on the shapes of erythrocyte doublets. Soft Matter.

[55] Shelby J.P., White J., Chiu D.T. (2003). A microfluidic model for single-cell capillary obstruction by Plasmodium falciparum-infected erythrocytes. Proc. Natl. Acad. Sci. USA.

[56] Jülich Supercomputing Centre (2021). JURECA: Data Centric and Booster Modules implementing the Modular Supercomputing Architecture at Jülich Supercomputing Centre. J. Large-Scale Res. Facil..

